# Brain uptake and safety of Flutemetamol F 18 injection in Japanese subjects with probable Alzheimer’s disease, subjects with amnestic mild cognitive impairment and healthy volunteers

**DOI:** 10.1007/s12149-017-1154-7

**Published:** 2017-02-08

**Authors:** Takami Miki, Hiroyuki Shimada, Jae-Seung Kim, Yasuji Yamamoto, Masakazu Sugino, Hisatomo Kowa, Kerstin Heurling, Michelle Zanette, Paul F. Sherwin, Michio Senda

**Affiliations:** 1 Department of Geriatrics, Osaka City University Hospital, 5-7, Asahi-machi 1-chome, Abeno-ku, Osaka City, Japan; 2 Nuclear Medicine Department, Asan Medical Center, 388-1 Pungnap-2 Dong, Songpa-Gu, Seoul, South Korea; 3 Neuropsychiatry Department, Kobe University Hospital, 5-2, Kusunoki-cho 7-chome, Chuo-ku, Kobe City, Hyogo Prefecture Japan; 4 Aino Hospital, Center of Geriatric Somato-Psychological Care, 11-18, Takada-cho, Ibaraki City, Osaka Japan; 5 Neurology Department, Kobe University Hospital, 5-2, Kusunoki-cho 7-chome, Chuo-ku, Kobe City, Hyogo Prefecture Japan; 6 GE Healthcare, Uppsala, Sweden; 7 Nuclear Medicine and PET, Uppsala University, Uppsala, Sweden; 8 GE Healthcare, Marlborough, MA USA; 9 Positron Medical Department, Institute of Biomedical Research and Innovation Hospital, 2, Minatojima Minami-machi 2-chome, Chuo-ku, Kobe City, Hyogo Prefecture Japan; 10 Izumiotsu Municipal Hospital, Shimojyo-chou 16-1, Izumiotsu, Osaka, 595-0027 Japan

**Keywords:** [^18^F]Flutemetamol, Alzheimer’s disease, Radiotracer, β-Amyloid

## Abstract

**Objective:**

This Phase 2 study assessed the performance of positron emission tomography (PET) brain images made with Flutemetamol F 18 Injection in detecting β-amyloid neuritic plaques in Japanese subjects.

**Methods:**

Seventy subjects (25 with probable Alzheimer’s disease (pAD), 20 with amnestic mild cognitive impairment (aMCI), and 25 cognitively normal healthy volunteers[HVs]) underwent PET brain imaging after intravenous Flutemetamol F 18 Injection (185 MBq). Images were interpreted as normal or abnormal for neuritic plaque density by each of five non-Japanese and five Japanese readers who were blinded to clinical data. The primary efficacy analysis (based on HV and pAD data) was the agreement of the non-Japanese readers’ image interpretations with the clinical diagnosis, resulting in estimates of positive percent agreement (PPA; based on AD subjects; similar to sensitivity) and negative percent agreement (NPA; based on HVs; similar to specificity). Secondary analyses included PPA and NPA for the Japanese readers; inter-reader agreement (IRA); intra-reader reproducibility (IRR); quantitative image interpretations (standardized uptake value ratios [SUVRs]) by diagnostic subgroup; test–retest variability in five pAD subjects; and safety.

**Results:**

PPA was 92% for all non-Japanese readers and ranged from 88 to 92% for the Japanese readers. NPA ranged from 96 to 100% for both the non-Japanese readers and the Japanese readers. The majority image interpretations (the interpretations made independently by ≥3 of 5 readers) resulted in PPA values of 92 and 92% and NPA values of 100 and 96% for the non-Japanese and Japanese readers, respectively. IRA and IRR were strong. Composite SUVR values (mean of multiple regional values) allowed clear differentiation between pAD subjects and HVs. Test–retest variability ranged from 1.14 to 2.27%, and test–retest agreement of the blinded visual interpretations was 100% for all readers. Flutemetamol F 18 Injection was generally well tolerated.

**Conclusions:**

The detection of brain neuritic plaques in Japanese subjects using [^18^F]Flutemetamol PET images gave results highly consistent with clinical diagnosis, with non-Japanese and Japanese readers giving similar results. Inter-reader agreement and intra-reader reproducibility were high for both sets of readers. Visual delineation of abnormal and normal scans was corroborated by quantitative assessment, with low test–retest variability.

**Trial registration:**

Clinicaltrials.gov registration number NCT02813070.

**Electronic supplementary material:**

The online version of this article (doi:10.1007/s12149-017-1154-7) contains supplementary material, which is available to authorized users.

## Introduction

The rapid growth of the aged population in Japan [1] poses medical and economic challenges because of age-associated diseases such as dementia, of which the prevalence increased significantly from 1985 to 2005 [2]. Alzheimer’s disease (AD) is the predominant type of dementia in the Japanese population, [1, 2] with an incidence rate comparable to that of Western populations [3]. The presence of amyloid plaques in the brain is one of the microscopic hallmarks of AD. While the presence of amyloid plaques is necessary but not sufficient for a pathological diagnosis of AD, an absence of plaques excludes AD. The amyloid plaques of AD result from aggregation of amyloid-beta (Aβ) peptides formed by secretase-catalyzed cleavage of amyloid precursor protein.

Although a definitive diagnosis of AD requires microscopic examination of brain tissue obtained at biopsy or autopsy, [4] recently approved amyloid-specific positron emission tomography (PET) radiotracers may facilitate in-life early detection or exclusion of amyloid plaques in a routine clinical setting. One of the first amyloid PET imaging agents was [^11^C]Pittsburgh compound B ([^11^C]PiB), and it is probably the most widely studied agent. Its molecular structure is similar to thioflavin T, with modifications to allow it to cross the blood brain barrier, resulting in excellent visualization of brain amyloid [5–9]. However, the short radioactive half-life of carbon-11 (~20 min) limits [^11^C]PiB’s use to centers with on-site cyclotrons [10]. Efforts to develop radiotracers using the longer-lived positron-emitting isotope fluorine-18 (radioactive *t*_1/2_ ~110 min) resulted in marketing authorization of three commercially available products: florbetapir, flutemetamol, and florbetaben. One of these, [^18^F]flutemetamol (Vizamyl™, GE Healthcare, Marlborough, MA), recently gained regulatory approval in the USA and Europe as a diagnostic drug, and in Japan as a medical device for imaging neuritic amyloid plaques in the brain. The chemical structure of [^18^F]flutemetamol is nearly identical to that of [^11^C]PiB, differing only by the presence of the fluorine atom.

Prior clinical studies showed a strong correlation between cortical brain uptake of [^18^F]flutemetamol and quantitative measures of amyloid burden, [11] an ability to detect brain amyloid comparable to that of [^11^C]PiB, [12] and excellent sensitivity and specificity for detecting/excluding amyloid [12–14]. The clinical development program that was the basis for US and European approvals enrolled 761 subjects. Of these, 27 (4%) were Asian, including 22 (14 healthy volunteers and 8 AD patients) that were enrolled in a Japanese Phase 1 study [15]. The Phase 2 study reported in this paper explored further the safety and efficacy of [^18^F]flutemetamol in a larger Japanese population which included healthy volunteers, patients with probable Alzheimer’s disease, and patients with mild cognitive impairment. A combined data set of 831 total subjects was the basis for approval in Japan. The design of the Phase 2 study in Japan was comparable to the design of the study performed for approval in the US and Europe and hence a comparison of these results of two studies was an important part of the Japanese approval process. Performance of an amyloid PET agent across different geographies is important to document so that data and studies can be used for registration in multiple territories. The pivotal studies presented in all countries has been the autopsy verification study in end-of-life subjects where the sensitivity and specificity of [^18^F]flutemetamol to detect β-amyloid in the brain were determined using neuropathologically determined neuritic plaque levels as the standard of truth [14]. In addition, two previous papers have described (a) the pharmacokinetics, biodistribution and internal radiation dosimetry profiles and (b) exploratory brain uptake of [^18^F]flutemetamol in Japanese subjects and have indicated that the molecule behaves comparably in small pilot populations [15–18]. The study reported here was the phase-II clinical trial of [^18^F]flutemetamol in Japan (GE-067-017) and it assessed the performance and safety of [^18^F]flutemetamol when studied in a larger population of elderly controls, Alzheimer’s disease and mild cognitive impairment patients.

## Materials and methods

### Objectives

The primary objective of this study was to assess the performance of Flutemetamol F 18 Injection in Japanese subjects as indicated by the level of agreement with clinical diagnosis of the blinded visual interpretations of [^18^F]flutemetamol brain images made by the non-Japanese readers. To determine if the performance of the tracer in Japanese subjects was comparable to its performance in other geographic territories, the images were interpreted by non-Japanese readers in the same way as in the previous studies in Europe and USA.

Secondary objectives included evaluation of agreement between Japanese and non-Japanese readers in their blinded visual interpretations of [^18^F]flutemetamol brain images; inter-reader agreement (IRA) and intra-reader reproducibility (IRR) of the blinded visual interpretation of [^18^F]flutemetamol brain images; the distributions and mean values of quantitative image interpretations (standardized uptake value ratios [SUVRs]) of [^18^F]flutemetamol brain images by the diagnostic subgroups (healthy volunteer [HV], amnestic mild cognitive impairment [aMCI], or pAD); test–retest variability in subjects with probable Alzheimer’s disease (pAD); association between SUVR and age in HVs; and safety of the drug product Flutemetamol F 18 Injection.

### Subjects

A total of six enrolling sites participated in the study, of which three sites also imaged their subjects and the other three sites had their subjects imaged in one of the imaging sites not far from theirs. At each participating center, the study protocol and informed consent form was approved by the ethics committee prior to subject screening and enrollment and the study was performed according to the standards of Good Clinical Practice and principles of the Declaration of Helsinki. Written informed consent was obtained from each subject prior to any study-related procedures. The study aimed to enroll 70 subjects of first-order Japanese descent with [^18^F]flutemetamol: 25 patients with pAD, 20 with aMCI, and 25 cognitively normal HVs (10 younger HVs aged 55 or less and 15 older HVs over age 55).

Each subject had at least 6 years of education, adequate visual, auditory and communication capabilities, and willingness and ability to comply with all study procedures, including standard tests of cognitive function. Each subject (and the caregiver, if relevant) was deemed by the investigator to be compliant and to have a high probability of completing the study. Women could not be of childbearing potential.

Subjects were excluded for unacceptable past radiation exposure; hypersensitivity to Flutemetamol F 18 Injection or any component; substance abuse; contraindication for MRI/PET; participation in a clinical trial of an investigational medicinal product within the past 30 days; positive serology for HBs, HCV, HIV, or syphilis; regular receipt of anticholinergic medication within the prior 3 months; and history of head injury that might interfere with the PET image interpretation.

pAD subjects were ≥55 years of age and met National Institute of Neurological and Communicative Diseases and Stroke–Alzheimer’s Disease and Related Disorders Association (NINCDS-ARDRA) criteria for pAD and the diagnostic and statistical manual of mental disorders-IV (DSM-IV) criteria for AD [19]. Other inclusion criteria for pAD subjects included: Mini Mental State Examination (MMSE^®^) score range of 15–26, clinical dementia rating scale (CDR) score of 0.5–2, a score of ≤4 on the Modified Hachinski Ischemic scale, brain MRI consistent with AD, and an appropriate caregiver capable of accompanying the subject on all study visits.

aMCI subjects were ≥55 years of age and met the Petersen criteria for aMCI [20]. Additional inclusion criteria were: MMSE of 27--30 and CDR of 0 or 0.5, a score of ≤4 on the Modified Hachinski Ischemic scale, brain MRI consistent with aMCI, and an appropriate caregiver capable of accompanying the subject on all study visits.

pAD and aMCI subjects were excluded for any of the following: a significant neurological or psychiatric disorder other than pAD that may affect cognition (including, but not limited to, major depression, schizophrenia, or mania); a previous history of clinically evident stroke, or significant cerebrovascular disease on brain imaging.

HVs were ≥25 years of age and had MMSE > 27, CDR 0, no signs of cognitive impairment, and a normal brain MRI. Exclusion criteria were: any clinically significant medical or neurological condition or any clinically significant abnormality on physical, neurological or laboratory examination; or a family history of pAD (more than one first degree relative with the diagnosis of pAD).

### Radiochemistry and imaging procedures

#### Tracer synthesis and administration

The investigational medicinal product Flutemetamol F 18 Injection was synthesized and handled according to Good Manufacturing Practice at two PET manufacturing sites, each located in the imaging site of this study, and was also transported to the third imaging site. Flutemetamol F 18 Injection was administered intravenously as a bolus dose (<40 s) via the antecubital vein. For subjects receiving a single dose of Flutemetamol F 18 Injection, the administered activity was 185 MBq, based on previous Phase I results [15–17]. In one enrolling site, 5 pAD subjects were enrolled in the test–retest cohort and each received two 120-MBq administrations of Flutemetamol F 18 Injection (for a cumulative total of 240 MBq).

#### MRI imaging

MRI was performed either on the screening day or at a separate visit but always prior to [^18^F]flutemetamol PET imaging to rule out cerebrovascular and structural disorders, as well as for volume-of-interest (VOI) analysis of the PET tracer uptake.

#### PET imaging

Three PET/CT scanners were used in the study: two GE Discovery 690 s and a Siemens Biograph 16. All scanners used iterative reconstruction and Gaussian post-reconstruction filtering to produce a net resolution of ~6 mm. Scanning started approximately 90 min following administration of Flutemetamol F 18 Injection, and lasted for 30 min. Data were collected as 5-min frames and were summed for visual assessment and quantitative analysis. In subjects who underwent two scans, the two scans were separated by 1–4 weeks and performed with the same scanner.

#### Image analysis

PET images were realigned to correct for inter-frame movement, summed to create a 30-min static image, and co-registered to the patient’s MRI. Each subject’s PET and MR images were spatially normalized to the ICBM152 [21] template space for definition of VOIs for quantitative image analysis using SUVR, where the uptake of tracer in a VOI was divided by the uptake in the cerebellar cortex (CER; primary reference region) or pons (alternative reference region). A composite SUVR was derived from the simple mean of the SUVRs of five anatomical regions outlined bilaterally (frontal cortex, anterior cingulate, parietal cortex, lateral temporal cortex, and posterior cingulate and precuneus).

#### Blinded image evaluation

The blinded visual interpretation of PET images was conducted by 10 independent physician readers (five non-Japanese and five Japanese) who were experienced in nuclear medicine image interpretation. Japanese readers had been trained and board certified in Japan and were practicing currently in Japan, and non-Japanese readers had been trained and board certified outside of Japan and were practicing currently outside Japan. To be qualified as a reader, each candidate was trained to assess images using GE’s interactive electronic training program for the interpretation of [^18^F]flutemetamol images including a classification test which had to be passed. The readers independently read and classified each study subject’s images as either normal or abnormal for neuritic plaque density in separate blinded image evaluation sessions for the non-Japanese and Japanese readers.

### Safety assessments

Subjects were monitored for adverse events (AEs) from the start of the first administration of study tracer up to 24 h afterward. Vital signs (temperature, pulse, respiration, blood pressure), 12-lead electrocardiograms, and clinical laboratory parameters were evaluated at pre-specified pre- and post-treatment time points. Each subject received a physical examination at screening and before and after scanning.

### Statistical analyses

All statistical analyses were performed using SAS^®^ software Version 9.2 (SAS Institute Inc., Cary, NC). For some analyses, HVs were stratified into two age groups; 10 younger HVs (25–55 years) and 15 older HVs (≥55 years old).

#### Primary efficacy analysis

The primary efficacy analysis (based on the data from HVs and pAD subjects) was the agreement of the non-Japanese readers’ image interpretations with clinical diagnosis (used in lieu of having histopathology as the SoT), resulting in estimates of positive percent agreement (PPA; similar to sensitivity; determined in patients with a clinical diagnosis of AD) and negative percent agreement (NPA; similar to specificity; determined in the HV subjects). Each image interpretation for each subject was compared to his/her clinical diagnosis and classified as an apparent True Positive (TP), True Negative (TN), False Positive (FP), or False Negative (FN) result, and the numbers of each classification (nTP, nTN, nFP, nFN) were determined for each reader and used to calculate PPA and NPA for each reader using the following formulas:

Positive Percent Agreement (PPA) = nTP/(nTP + nFN).

Negative Percent Agreement (NPA) = nTN/(nFP + nTN).

Majority of image interpretations were determined from image interpretation made independently by the majority (i.e., at least 3) of 5 readers in the reader group (non-Japanese or Japanese) being analyzed. For example, if 3, 4, or 5 of the readers independently interpreted a subject’s PET image as “normal”, then the majority interpretation of that image was “normal”. Majority of image interpretations were classified as TP, TN, FP, or FN and majority values for PPA and NPA were determined as described above.

#### Inter-reader agreement (IRA)

Pair-wise IRA of blinded visual image interpretation was determined as Cohen’s kappa, [22] and classified as excellent (>0.9), very good (>0.8 and ≤0.9), or good (>0.7 and ≤0.8). Agreement across all non-Japanese readers and Japanese readers was determined as Fleiss’ kappa.

#### Intra-reader reproducibility (IRR)

IRR was measured as the percentage of images for which a reader’s second interpretation of an image agreed with the reader’s first interpretation of the image. This was determined using duplicate images for seven subjects (approximately10%) drawn randomly from the 65 subjects who received single administrations of Flutemetamol F 18 Injection. The duplicate images were inserted randomly into the image set interpretated by each reader.

#### Quantitative assessment—SUVR

SUVR measurements of [^18^F]flutemetamol brain images were used to quantify brain uptake of the tracer. SUVR was defined as the ratio of each target region’s standardized uptake value (SUV) to the SUV in a reference region; this was calculated using internally developed software. SUVs for the target and reference regions were obtained from the VOI values by normalizing average tissue concentration in the VOI by the injected administration and weight of the subject using the following formula:

SUV = Measured Activity Concentration in VOI / Injected activity / Weight of subject.

SUVR was determined separately for the cerebellar cortex (SUVR-CER) and the pons (SUVR-PONS) as reference regions. Composite SUVR values representing all regions analyzed were calculated by simple averaging of the SUVR values for the anterior cingulate, frontal cortex, parietal cortex, lateral temporal cortex and precuneus/posterior cingulate regions.

#### Calculation of optimal thresholds

The mean and standard deviation (SD) for SUVR were calculated region-wise for the pAD group and HV group separately. The data were checked for outliers and none were identified.

In each region, the optimal SUVR threshold (OSUVRT) was defined as the SUVR that resulted in the maximum percentage of correctly classified HV and pAD subjects. The OSUVRTs were calculated by locating the exact midpoint expressed in SDs between the mean SUVRs of the pAD and HV groups:

OSUVRT = [meanpAD − (factor × SDpAD)].

where:

factor = [meanpAD − meanHV] / [SDpAD + SDHV].

#### Determination of PPA and NPA Using SUVR values

Composite SUVR-CER values for pAD and HV subjects were classified as abnormal (positive) if they were above the optimal SUVR threshold and normal (negative) if they were at or below the optimal SUVR threshold. The SUVR-CER classifications were compared to clinical diagnosis and sub-classified as TN, TP, FP, or FN, and the numbers of each sub-classification were used to calculate PPA and NPA, which are reported with exact binomial 95% confidence intervals.

#### Test–retest variability

Test–retest variability (TRV) was determined for five pAD subjects who each received two 120-MBq administrations of Flutemetamol F 18 Injection and subsequent PET scans (1–4 weeks apart). Percent TRV was calculated as the absolute value of the difference between the first (test) value and the second (retest) value divided by the mean and multiplied by 100 percent:

%TRV = 100% × | SUVR_1 −_SUVR_2_|/((SUVR_1_ + SUVR_2_)/2).

In the case of perfect agreement, SUVR_1_ and SUVR_2_ would be equal, and %TRV would be 0%, indicating no variability. The variability estimate was calculated for each subject, for each brain VOI and the composite measure.

#### Association between SUVR and Age

The association between the composite SUVR and the age of HV subjects was determined by Pearson’s correlation (*r*) and a regression model of the SUVR as the dependent variable and subject age as the independent variable.

#### Classification of aMCI Subjects’ Images

The classification of aMCI subjects’ images as normal or abnormal is presented descriptively with number and percentage. This assessment was provided based on both visual interpretation and composite SUVR compared to the optimal regional thresholds.

## Results

In total, 87 Japanese subjects (28 pAD, 23 aMCI and 36 HVs) at six centers (five in Japan, one in Korea) signed informed consent and were enrolled in this study; 17 withdrew before dosing, 13 due to screen failure. Seventy subjects received Flutemetamol F 18 Injection, and all 70 completed the study and were included in both the efficacy and safety populations. Demographic and baseline neuropsychological data are summarized in Table 1.

**Table 1:** Summary of Subject Demographics and Baseline Neuropsychological Status – Safety Population

Variable	Statistics/category	Clinical diagnosis at screening					Total *N* = 70
		Probable AD *N* = 25	Amnestic MCI *N* = 20	Healthy Volunteer			
				≤55 years *N* = 10	>55 years *N* = 15	All HV *N* = 25	
Age (years)^a^	*n*	25	20	10	15	25	70
	Mean (SD)	75 (6)	71 (7)	50 (8)	63 (6)	57 (9)	68 (11)
	≤55 years, n (%)	0 (0)	0 (0)	10 (100)	0 (0)	10 (40)	10 (14)
	>55 years, n (%)	25 (100)	20 (100)	0 (0)	15 (100)	15 (60)	60 (86)
Gender, *n* (%)	Male	9 (36)	11 (55)	6 (60)	8 (53)	14 (56)	34 (49)
	Female	16 (64)	9 (45)	4 (40)	7 (47)	11 (44)	36 (51)
Race, *n* (%)	Japanese	25 (100)	20 (100)	10 (100)	15 (100)	25 (100)	70 (100)
Height (cm)	*n*	25	20	10	15	25	70
	Mean (SD)	156 (7)	158 (10)	165 (7)	163 (10)	163 (9)	159 (9)
Weight (kg)	*n*	25	20	10	15	25	70
	Mean (SD)	53 (10)	56 (9)	66 (11)	59 (9)	62 (11)	57 (11)
BMI (kg/m^2^)	*n*	25	20	10	15	25	70
	Mean (SD)	22 (3)	23 (3)	25 (4)	22 (3)	23 (3)	23 (3)
MMSE	*n*	25	20	10	15	25	70
	Mean (SD)	21.1 (3.07)	28.4 (0.81)	30.0 (0.00)	29.9 (0.35)	29.9 (0.28)	26.3 (4.40)
	95% CI	19.8, 22.3	28.0, 28.7	NC	29.7, 30.1	29.8, 30.0	25.3, 27.4
Modified Hachinski Ischemic Scale	*n*	25	20	NA	NA	NA	45
	Mean (SD)	0.7 (0.69)	0.8 (0.70)	–	–	–	0.7 (0.69)
	95% CI	0.4, 1.0	0.5, 1.1	–	–	–	0.5, 0.9
CDR	*n*	25	20	10	15	25	70
	Mean (SD)	0.94 (0.391)	0.38 (0.222)	0.00 (0.000)	0.00 (0.000)	0.00 (0.000)	0.44 (0.478)
	95% CI	0.78, 1.10	0.27, 0.48	NC	NC	NC	0.33, 0.56

*AD* Alzheimer’s disease, *BMI* body mass index, *CDR* Clinical Dementia Rating, *DSM-IV* Diagnostic and Statistical Manual of Mental Disorders, 4th Edition, *HV* healthy volunteer, *MCI* mild cognitive impairment, *MMSE* Mini-Mental State Examination, *N* safety population, *n* number of subjects in category, *NA* not applicable, *NC* unable to be calculated, *NINCDS-ADRDA* National Institute of Neurological and Communicative Disorders and Stroke-Alzheimer’s Disease and Related Disorders Association, *SD* standard deviation, %, 100% × n/N

^a^Age was calculated as [Date of Informed Consent—Date of Birth] / 365.25 rounded down to the nearest integer

Among the non-Japanese readers, PPA was 92% (95% CI, 74, 99) for all readers and NPA ranged from 96% (95% CI, 80%, 100%) to 100% (95% CI, 86%, 100%) (median, 100%; majority 100% [95% CI, 86%, 100%]). The area under the reader performance curve (analogous to a receiver operating characteristic [ROC] curve) was 0.96, close to that of a perfect test, which would have an area of 1.

**Fig. 1:**
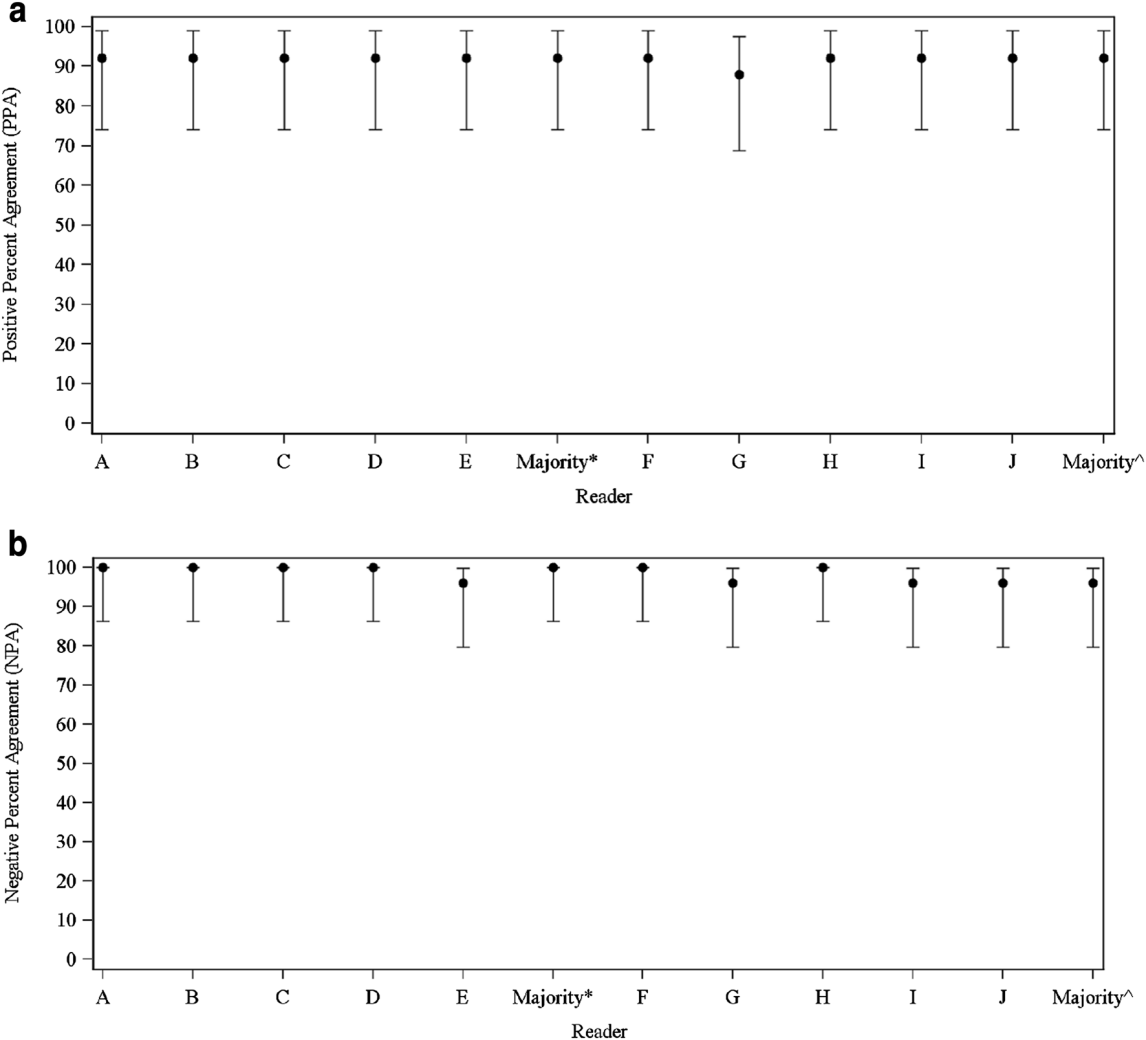
Blinded Visual Interpretations for Non-Japanese and Japanese Readers—efficacy population. **a** positive percent agreement. **b** Negative percent agreement. The analyses are based on blinded visual interpretations of the images collected after the first dose of Flutemetamol F 18 Injection. *Error bars* represent 95% exact binomial confidence interval. *Majority interpretation by non-Japanese readers (Readers *A, B, C, D* and *E*). ^Majority interpretation by Japanese readers (Readers *F, G, H, I* and *J*)

Among the Japanese readers, PPA ranged from 88% (95% CI, 69, 98%) to 92% (95% CI, 74, 99%) (median, 92%; majority 92% [95% CI, 74, 99%]) and NPA ranged from 96% (95% CI, 80, 100%) to 100% (95% CI, 86, 100%) (median, 96%; majority 96% [95% CI, 80, 100%]). The area under the reader performance curve is 0.96. PPA and NPA for Japanese and non-Japanese readers were comparable (Fig. 1).

IRA values **(**Table 2, S1**)** showed high levels of agreement across readers. Among the non-Japanese readers, percentage agreement for reader pairs ranged from 95 to 100%, with a median of 99%. Cohen’s kappa scores (95% CI) ranged from 0.91 (0.80, 1.00) to 1.00 (1.00, 1.00), with a median of 0.97 (0.91, 1.00). Across all five non-Japanese readers, there was complete agreement for 95% of images read; Fleiss’ kappa (95% CI) was 0.96 (0.89, 1.00).

**Table 2:** Summary of Inter-reader agreement (ira)—efficacy population

Comparison	Statistic	Percent	Kappa (95% CI)
Non-Japanese to Non-Japanese	Min	95	0.91 (0.80, 1.00)
	Max	100	1.00 (1.00, 1.00)
	Median	99	0.97 (0.91, 1.00)
Japanese to Japanese	Min	95	0.91 (0.80, 1.00)
	Max	99	0.97 (0.91, 1.00)
	Median	97	0.94 (0.85, 1.00)
Non-Japanese to Japanese	Min	94	0.88 (0.76, 0.99)
	Max	100	1.00 (1.00, 1.00)
	Median	97	0.94 (0.85, 1.00)

Among the Japanese readers, percentage agreement for reader pairs ranged from 95 to 99%, with a median of 97%. Cohen’s kappa scores (95% CI) ranged from 0.91 (0.80, 1.00) to 0.97 (0.91, 1.00), with a median of 0.94 (0.85, 1.00). Across all five Japanese readers, there was complete agreement for 94% of images read; Fleiss’ kappa (95% CI) was 0.94 (0.86, 1.00). IRR (Table 3) was 7/7 (100%) for four of the five readers in each group, and was 6/7 (86%) for the remaining reader in each group.

**Table 3:** Summary of intra-reader reproducibility (IRR)—efficacy population

Reader	IRR, *n* (%)
Non-Japanese	
A, C, D, E	7 (100)
B	6 (86)
Japanese	
G, H, I, J	7 (100)
F	6 (86)

Quantitative analysis by SUVR (Table 4) showed some clear trends. The ordering of mean regional and composite SUVR values, from highest to lowest values, was: pAD > MCI > EHV > YHV, consistent with the known association of amyloid burden and diagnosis. Because of the large difference in SUVR values, there was clear differentiation between subjects with pAD and HVs, in all cortical regions and in the composite VOI. There was less differentiation between subjects with pAD and those with aMCI, related to the smaller differences in SUVR. Despite the smaller differences, none of the 95% CIs for the pAD and the aMCI subjects overlapped, indicating a statistically meaningful difference (although no formal hypothesis test was performed) between the mean SUVRs. Within each cohort, the order of SUVR values, from highest to lowest, was: posterior cingulate > anterior cingulate > lateral temporal > parietal ≈ frontal. Results using mean SUVR-PONS values were similar to those using SUVR-CER (data not shown).

**Table 4:** Summary of SUVR by region and clinical diagnosis for cerebellum reference region—efficacy population

First dose of Flutemetamol F 18 injection (185 MBq)							
Variable	Statistics/category	Clinical diagnosis at screening					Total *N* = 65
		Probable AD *N* = 20	Amnestic MCI *N* = 20	Healthy volunteer			
				≤55 years	>55 years	All HV	
				*N* = 10	*N* = 15	*N* = 25	
Anterior cingulate cortex	*n*	20	20	10	15	25	65
	Mean (SD)	2.19 (0.518)	1.71 (0.332)	1.15 (0.084)	1.29 (0.134)	1.23 (0.133)	1.68 (0.528)
	95% CI	1.95, 2.43	1.56, 1.87	1.09, 1.21	1.21, 1.36	1.18, 1.29	1.54, 1.81
Frontal cortex	*n*	20	20	10	15	25	65
	Mean (SD)	1.95 (0.429)	1.52 (0.301)	1.08 (0.066)	1.13 (0.089)	1.11 (0.083)	1.49 (0.454)
	95% CI	1.75, 2.15	1.38, 1.66	1.04, 1.13	1.084, 1.182	1.08, 1.15	1.38, 1.61
Lateral temporal cortex	*n*	20	20	10	15	25	65
	Mean (SD)	1.98 (0.370)	1.56 (0.250)	1.17 (0.068)	1.27 (0.073)	1.23 (0.085)	1.56 (0.401)
	95% CI	1.81, 2.15	1.44, 1.68	1.12, 1.22	1.23, 1.31	1.19, 1.26	1.46, 1.66
Parietal cortex	*n*	20	20	10	15	25	65
	Mean (SD)	1.88 (0.366)	1.51 (0.282)	1.09 (0.039)	1.18 (0.094)	1.15 (0.088)	1.48 (0.399)
	95% CI	1.71, 2.05	1.38, 1.64	1.07, 1.12	1.13, 1.24	1.11, 1.18	1.39, 1.58
Posterior cingular cortex	*n*	20	20	10	15	25	65
	Mean (SD)	2.27 (0.487)	1.76 (0.370)	1.18 (0.079)	1.32 (0.122)	1.26 (0.126)	1.72 (0.541)
	95% CI	2.04, 2.50	1.58, 1.93	1.12, 1.24	1.25, 1.39	1.21, 1.32	1.59, 1.86
Composite VOI ^a^	*n*	20	20	10	15	25	65
	Mean (SD)	2.05 (0.424)	1.61 (0.297)	1.13 (0.056)	1.24 (0.082)	1.20 (0.088)	1.59 (0.459)
	95% CI	1.86, 2.25	1.47, 1.75	1.09, 1.18	1.19, 1.28	1.16, 1.23	1.48, 1.70
First dose of Flutemetamol F 18 injection (all subjects)							
Variable	Statistics/category	Clinical diagnosis at screening					Total *N* = 70
		Probable AD *N* = 25	Amnestic MCI *N* = 20	Healthy volunteer			
				≤55 years	>55 years	All HV	
				*N* = 10	*N* = 15	*N* = 25	
Anterior cingulate cortex	n	25	20	10	15	25	70
	Mean (SD)	2.20 (0.466)	1.71 (0.332)	1.15 (0.084)	1.29 (0.134)	1.23 (0.133)	1.72 (0.530)
	95% CI	2.01, 2.39	1.56, 1.87	1.09, 1.21	1.21, 1.36	1.18, 1.29	1.59, 1.84
Frontal cortex	*n*	25	20	10	15	25	70
	Mean (SD)	1.96 (0.393)	1.52 (0.301)	1.08 (0.066)	1.13 (0.089)	1.11 (0.083)	1.53 (0.459)
	95% CI	1.80, 2.12	1.38, 1.66	1.04, 1.13	1.08, 1.18	1.08, 1.15	1.42, 1.64
Lateral temporal cortex	*n*	25	20	10	15	25	70
	Mean (SD)	2.00 (0.348)	1.56 (0.250)	1.17 (0.068)	1.267 (0.073)	1.23 (0.085)	1.60 (0.412)
	95% CI	1.85, 2.14	1.44, 1.68	1.12, 1.22	1.23, 1.31	1.19, 1.26	1.50, 1.70
Parietal cortex	*n*	25	20	10	15	25	70
	Mean (SD)	1.90 (0.348)	1.51 (0.282)	1.09 (0.039)	1.18 (0.094)	1.15 (0.088)	1.52 (0.412)
	95% CI	1.76, 2.05	1.38, 1.64	1.07, 1.12	1.13, 1.24	1.11, 1.18	1.42, 1.62
Posterior cingular cortex	*n*	25	20	10	15	25	70
	Mean (SD)	2.28 (0.451)	1.76 (0.370)	1.18 (0.079)	1.32 (0.122)	1.26 (0.126)	1.77 (0.548)
	95% CI	2.09, 2.46	1.58, 1.93	1.12, 1.24	1.25, 1.39	1.21, 1.32	1.64, 1.90
Composite VOI ^1^	*n*	25	20	10	15	25	70
	Mean (SD)	2.07 (0.390)	1.61 (0.297)	1.13 (0.057)	1.24 (0.082)	1.20 (0.088)	1.63 (0.466)
	95% CI	1.91, 2.23	1.47, 1.75	1.09, 1.18	1.19, 1.28	1.16, 1.23	1.52, 1.74

*AD* Alzheimer’s disease, *CI* confidence interval, *HV* healthy volunteer, *MCI* mild cognitive impairment, *N* efficacy population, *n* number of subjects in category, *NA* not applicable, *SD* standard deviation, *SUVR* standardized uptake value ratio, *VOI* volume of interest

^a^Composite VOI determined from the anterior cingulate, frontal cortex, parietal cortex, lateral temporal cortex and a VOI covering precuneus and posterior cingulate

The OSUVRTs for cerebellum and pons (OSUVRT-CER and OSUVRT-PONS, respectively) for the composite VOI were 1.357 and 0.596, respectively. Using OSUVRT-CER, PPA and NPA were 96% (95% CI, 80, 100%) and 88% (95% CI 69, 98%), respectively. Using OSUVRT-PONS, PPA and NPA were 92% (95% CI 74, 99%) and 92% (95% CI 74, 99%), respectively. There was 85–100% of agreement between the visual image classifications by majority read assessment and the classification based on the optimal SUVR threshold.

For the 5 subjects with pAD who received a second dose of Flutemetamol F 18 Injection, SUVR-CER values following the second dose were similar to those seen following the first dose (data not shown), resulting in %TRV values that ranged from 1.85 to 2.27% for SUVR-CER and 1.14–2.11% for SUVR-PONS. Test–retest agreement of the blinded visual interpretations was 100% for each of the ten readers.

**Fig. 2:**
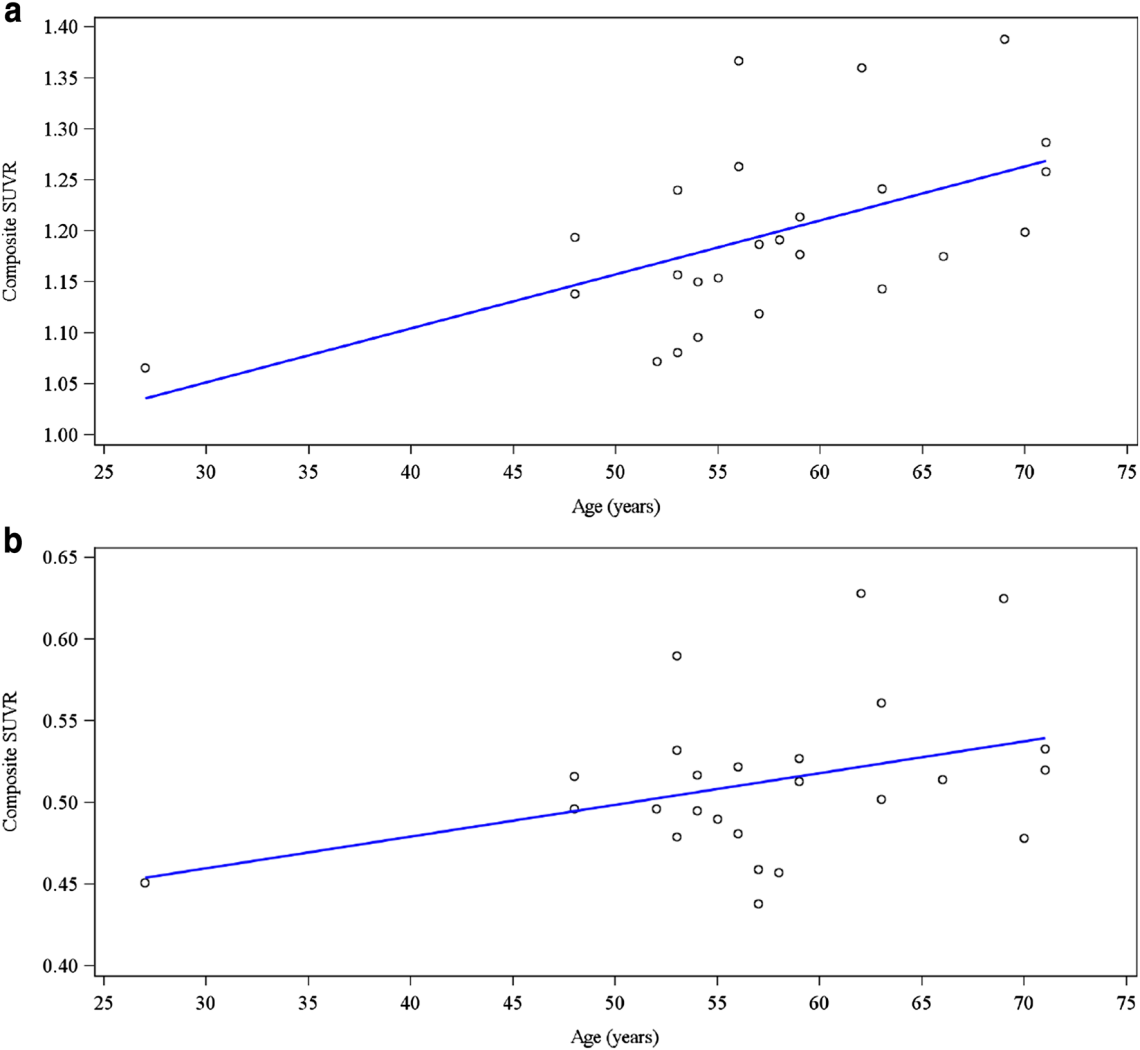
Composite SUVR Values and Age for HV subjects (efficacy population) [Clinical diagnosis at screening as HV (*N* = 25)] Using **a** cerebellum reference region (SUVR-CER), and **b** Pons Reference Region (SUVR-PONS). For the cerebellum reference region, the regression line was plotted based on SUVR = 0.8931 + 0.0053 *AGE with *R* ^2^ = 0.3055. For the pons reference region, the regression line was plotted based on SUVR = 0.4013 + 0.0019 *AGE with *R* ^2^ = 0.1392. HV, healthy volunteer; SUVR, standardized uptake value ratio.

The trend for higher SUVR values in older subjects evident in Table 4 was confirmed and quantified through correlation analysis. There was significant correlation between SUVR-CER and age, with a Pearson correlation coefficient of 0.5527 (*p* = 0.0042, Fig. 2). A regression model confirmed the correlation between SUVR-CER and age, with *R* ^2^ = 0.3055. The correlation between SUVR-PONS and age approached but did not achieve statistical significance (Pearson correlation coefficient 0.3731 [*p* = 0.0662], *R* ^2^ = 0.1392).

Table 5 summarizes the image interpretations for the 20 aMCI subjects. Approximately half (45–55%) were assigned to each category. Based on the optimal SUVR threshold classification, 13 (65%) subjects’ scans were abnormal and 7 (35%) were normal using the optimal SUVR-CER threshold classification, and 10 (50%) subjects’ scans were abnormal and 10 (50%) were normal using the optimal SUVR-PONS threshold classification.

**Table 5:** Summary of determination of aMCI subjects—efficacy population

Assessment	*N* = 20	
	Abnormal (positive) *n* (%)	Normal (negative) *n* (%)
Blinded visual read (non-Japanese readers)		
Reader A	9 (45)	11 (55)
Reader B	10 (50)	10 (50)
Reader C	9 (45)	11 (55)
Reader D	9 (45)	11 (55)
Reader E	10 (50)	10 (50)
Blinded visual read (Japanese readers**)**		
Reader F	11 (55)	9 (45)
Reader G	10 (50)	10 (50)
Reader H	11 (55)	9 (45)
Reader I	11 (55)	9 (45)
Reader J	11 (55)	9 (45)
Optimal SUVR-CER threshold classification^a^	13 (65)	7 (35)
Optimal SUVR-PONS threshold classification^a^	10 (50)	10 (50)

*aMCI* amnestic mild cognitive impairment, *HV* healthy volunteer, *N* efficacy population, *n* number of subjects in category, *pAD* probable Alzheimer’s disease, *SUVR* standardized uptake value ratio, *SUVR-CER*, SUVR based on the cerebellum as reference region, *SUVR-PONS*, SUVR based on the pons as reference region

^a^The optimal SUVR threshold was calculated as: SUVR^OT,^ [mean_pAD_ − factor x SD_pAD_], where factor, [mean_pAD_ – mean_HV_] / [SD_pAD_ + SD_HV_]. If SUVR-CER > 1.357 or SUVR-PONS > 0.596, the SUVR was defined as “abnormal.”

**Fig. 3:**
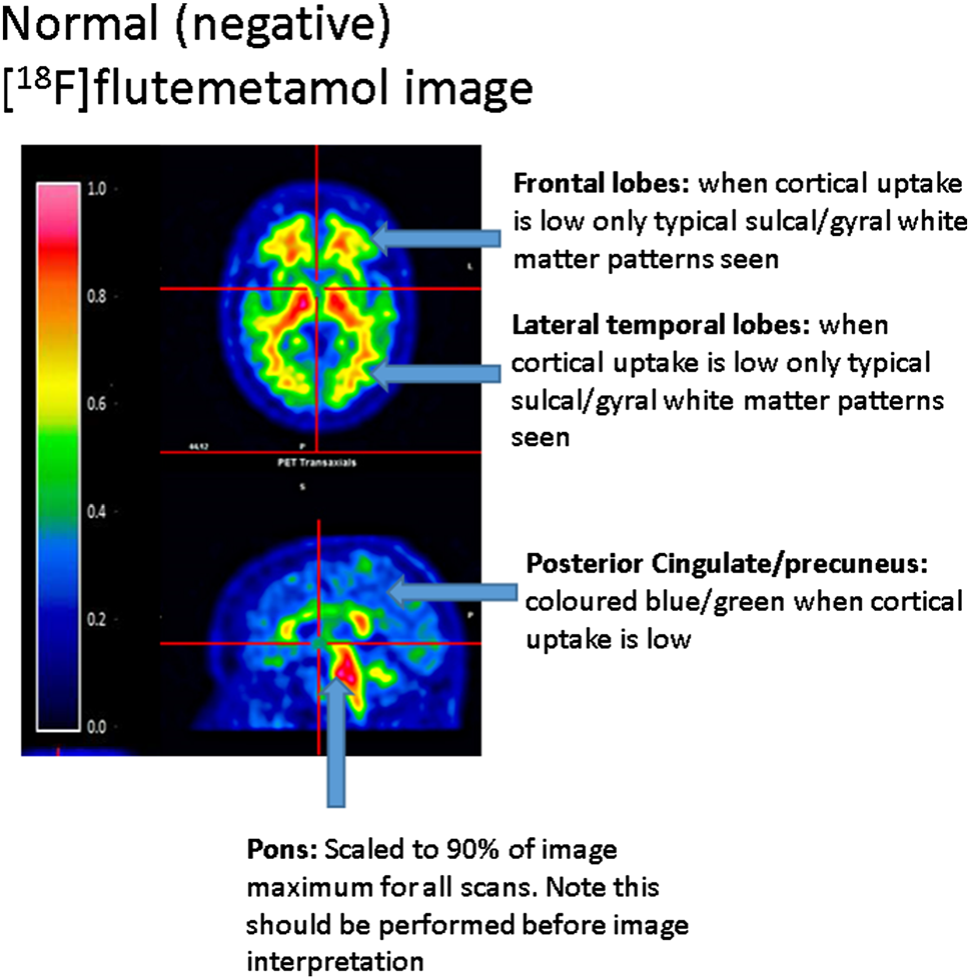
Example negative images

**Fig. 4:**
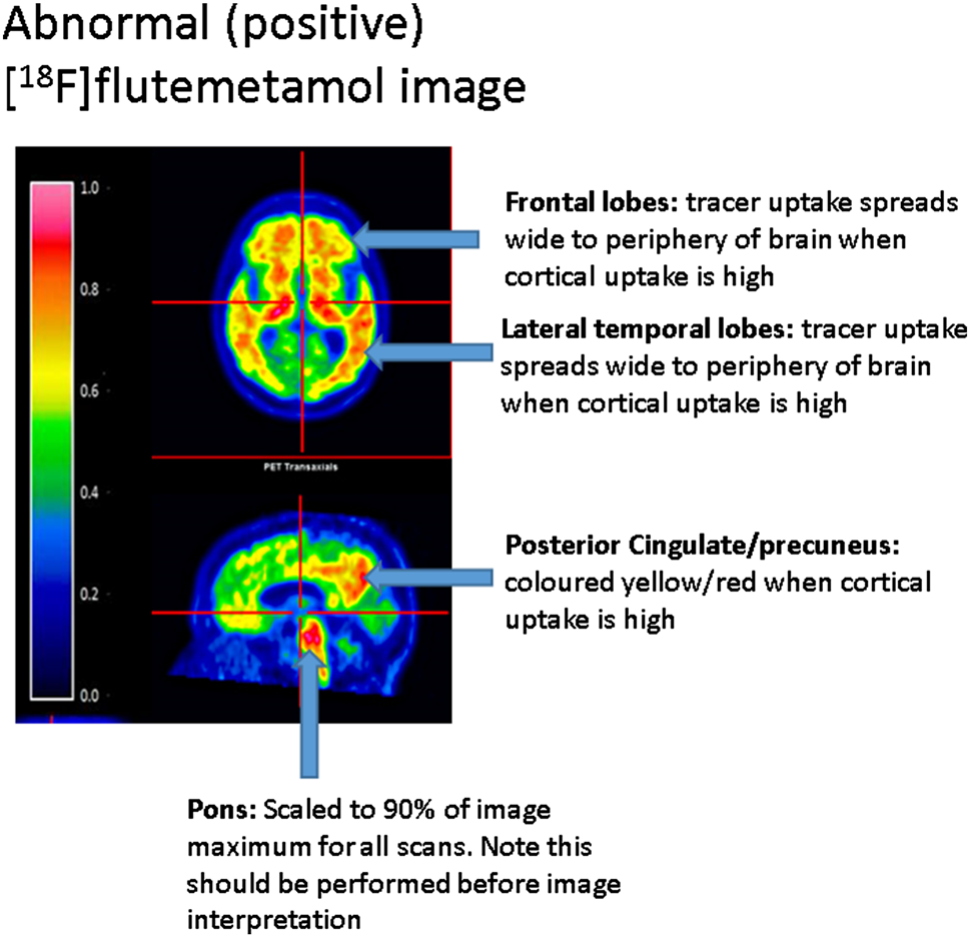
Example positive images

Example images are provided in Fig. 3 (negative) and Fig. 4 (positive).

### Safety

Single and repeat doses of Flutemetamol F 18 Injection were generally well-tolerated by HVs and subjects with pAD and aMCI. AEs were reported in seven subjects (10%; Table 6). Two subjects (both HVs >55 years) experienced AEs that were deemed related to Flutemetamol Injection by the investigator: one subject experienced epigastric discomfort, flushing and hypertension, and another subject experienced headache.

**Table 6:** Overall summary of adverse events

	Clinical diagnosis at screening					
	Healthy volunteer					
	pAD *N* = 25 *n* (%)	aMCI *N* = 20 *n* (%)	≤55 years*N* = 10 *n* (%)	>55 years *N* = 15 *n* (%)	All HV *N* = 25 *n* (%)	Total *N* = 70 *n* (%)
Number of AEs reported	2	2	0	5	5	9
Subjects with any AE	2 (8)	2 (10)	0 (0)	3 (20)	3 (12)	7 (10)
Subjects with any AE at least possibly related to Flutemetamol F 18 injection	0 (0)	0 (0)	0 (0)	2 (13)	2 (8)	2 (3)
Subjects with AEs by intensity^a^						
Mild	2 (8)	2 (10)	0 (0)	3 (20)	3 (12)	7 (10)
Moderate	0 (0)	0 (0)	0 (0)	0 (0)	0 (0)	0 (0)
Severe	0 (0)	0 (0)	0 (0)	0 (0)	0 (0)	0 (0)
Subjects with Any Serious AE	0 (0)	0 (0)	0 (0)	0 (0)	0 (0)	0 (0)
Subjects with any serious AE at least possibly related to Flutemetamol F 18 injection	0 (0)	0 (0)	0 (0)	0 (0)	0 (0)	0 (0)
Subjects with any AE leading to study discontinuation	0 (0)	0 (0)	0 (0)	0 (0)	0 (0)	0 (0)
Subjects with any AE leading to death	0 (0)	0 (0)	0 (0)	0 (0)	0 (0)	0 (0)
Subjects with any ae leading to death at least possibly related to Flutemetamol F 18 injection	0 (0)	0 (0)	0 (0)	0 (0)	0 (0)	0 (0)

*AEs* adverse events, *aMCI* amnestic mild cognitive impairment, *HV* healthy volunteers, *N* safety population, *n* number of subjects in category, *pAD* probable Alzheimer’s disease, *AE* adverse event, %, 100% × n/N. Subjects reporting more than one event in a category are counted only once for that category

^a^Subjects reporting more than one event are counted only once at the highest intensity reported

All AEs were mild and all events resolved. There were no deaths, serious AEs, or withdrawals due to AEs. No clinically significant changes were reported in laboratory parameters, ECG, neurological or physical examinations. One subject had a clinically significant change in blood pressure that was mild in intensity and resolved, and was judged to be unrelated to the administration of Flutemetamol F 18 Injection.

## Discussion

This Phase 2, multicenter study in three groups of Japanese subjects (HVs, aMCI, and pAD) showed high levels of agreement between the subject’s clinical diagnosis and the blinded visual interpretation of [^18^F]flutemetamol brain images, as indicated by the high values for PPA (analogous to sensitivity) and NPA (analogous to specificity). The area under the reader performance curve was 0.96 for both groups of readers, indicating identical and nearly perfect overall performance; this is also evident from the nearly complete overlap in the 95% confidence intervals across the two groups of readers **(**Fig. 1 **)**. Two (8%) of the 25 pAD subjects diagnosed by clinical criteria were [^18^F]flutemetamol negative, whereas none of the HVs were deemed to have abnormal[^18^F]flutemetamol uptake above threshold values either by visual inspection or by quantitative means. Fleiss’ kappa scores near one indicated excellent IRA, and eight of ten readers had IRRs of 100%.

The efficacy of Flutemetamol F 18 Injection in this study is consistent with the results of a previously reported Phase 2 study in Western subjects [12]. In that study, only two out of 27 of the probable AD subjects had a negative scan indicating that expert clinical diagnosis was a useful surrogate for neuropathology as a standard of truth to calculate positive percent agreement. The term positive percent agreement was used in this Japanese study instead of sensitivity as this Japanese study used clinical diagnosis as the standard of truth as opposed to neuropathology which was used for sensitivity measurements in the pivotal phase III study described by Curtis et al. [14] These results were expected given the lack of ethnic differences in the density and distribution of hallmark lesions of AD, [23] and agreement on clinical diagnoses of dementia and dementia subtypes in Japanese and western populations when the American Psychiatric Association’s Diagnostics and Statistics Manual criteria were used [24]. The demonstration that the [^18^F]flutemetamol drug product performs comparably in both the Phase I and Phase II studies allowed the development program to use the Curtis study [14] autopsy patients as the pivotal data set for the registration of Flutemetamol F 18 Injection in Japan.

The main strength of this study is its comparisons of image interpretations made by Japanese readers relatively new to flutemetamol to those of more experienced readers, which showed that after a short training session, the Japanese image readers were as good as the non-Japanese readers.

The main weaknesses of this study include lack of a true standard of truth based on brain examination (which was considered unnecessary for the study goals), and use of younger HVs, which may have made it easier to differentiate between AD and HV subjects. However, because there was no histological standard of truth, the use of younger HVs may have helped avoid including cognitively normal HVs with asymptomatic brain amyloid, which may have confounded the results.

Test–retest reproducibility is an important determinant of the utility of an assay for longitudinal within-subject studies. The variability in test–retest SUVR was very low (less than 2.3%), and agreement in visual interpretations among readers was excellent. Overall, the test–retest variability of [^18^F]flutemetamol scans in pAD patients was similar between the Japanese and Western subjects as reported by Vandenberghe et al. [12].

As expected from results with other tracers such as [^11^C]PiB, mean SUVR was consistently lower in HVs compared to the patients with aMCI or pAD. Moreover, the range of SUVR values in Japanese HVs and cases with pAD overlapped with the range reported previously in the Western HVs and cases with pAD, respectively [12]. Subjects with aMCI demonstrated an SUVR distribution similar to that observed in Western subjects with MCI (i.e., a roughly 50:50 split between normal and abnormal scans). Strong agreement between quantitation (SUVR) and visual image classification was observed. Based on the absence of significant AEs related to Flutemetamol F 18 Injection, the results suggest that Flutemetamol F 18 Injection is safe and generally well tolerated by Japanese HVs and subjects with pAD and aMCI.

In conclusion, [^18^F]flutemetamol uptake allowed differentiation between Japanese patients with pAD and younger healthy controls. The results are similar to those reported in the Western population, giving no evidence of specific ethnic differences in the efficacy or safety of Flutemetamol F 18 Injection. After a short training period, Japanese readers were as proficient as more experienced non-Japanese readers. Overall, the results from this study show that Flutemetamol F 18 Injection is a robust tracer for in vivo detection of an increased brain β-amyloid load.

## Electronic supplementary material

Below is the link to the electronic supplementary material.

Supplementary material 1 (DOCX 40 KB)
