# Supplementary material for: Brain uptake and safety of Flutemetamol F 18 injection in Japanese subjects with probable Alzheimer’s disease, subjects with amnestic mild cognitive impairment and healthy volunteers
Source: Ann Nucl Med. 2017 Feb 8;31(3):260–72. doi: 10.1007/s12149-017-1154-7 (PMC5352784; doi:10.1007/s12149-017-1154-7)
Supplement: Supplementary file 1 — Supplementary material 1 (DOCX 40 KB) [file 12149_2017_1154_MOESM1_ESM.docx]

| Supplemental Data  Table S1. Summary of Inter-Reader Agreement – Efficacy Population | | | |
| --- | --- | --- | --- |
| **Reader Comparison^a^** | **Interpretations With Agreement Between Readers, n (%)** | **Kappa Statistic^b^ (95% CI)** | **Classification^c^** |
| A vs B | 64 (99) | 0.97 (0.91, 1.00) | Excellent |
| A vs C | 65 (100) | 1.00 (1.00, 1.00) | Excellent |
| A vs D | 65 (100) | 1.00 (1.00, 1.00) | Excellent |
| A vs E | 63 (97) | 0.94 (0.85, 1.00) | Excellent |
| A vs F | 63 (97) | 0.94 (0.85, 1.00) | Excellent |
| A vs G | 63 (97) | 0.94 (0.85, 1.00) | Excellent |
| A vs H | 63 (97) | 0.94 (0.85, 1.00) | Excellent |
| A vs I | 62 (95) | 0.91 (0.80, 1.00) | Excellent |
| A vs J | 62 (95) | 0.91 (0.80, 1.00) | Excellent |
| B vs C | 64 (99) | 0.97 (0.91, 1.00) | Excellent |
| B vs D | 64 (99) | 0.97 (0.91, 1.00) | Excellent |
| B vs E | 62 (95) | 0.91 (0.80, 1.00) | Excellent |
| B vs F | 62 (95) | 0.91 (0.80, 1.00) | Excellent |
| B vs G | 62 (95) | 0.91 (0.80, 1.00) | Excellent |
| B vs H | 62 (95) | 0.91 (0.80, 1.00) | Excellent |
| B vs I | 61 (94) | 0.88 (0.76, 0.99) | Very Good |
| B vs J | 63 (97) | 0.94 (0.85, 1.00) | Excellent |
| C vs D | 65 (100) | 1.00 (1.00, 1.00) | Excellent |
| C vs E | 63 (97) | 0.94 (0.85, 1.00) | Excellent |
| C vs F | 63 (97) | 0.94 (0.85, 1.00) | Excellent |
| C vs G | 63 (97) | 0.94 (0.85, 1.00) | Excellent |
| C vs H | 63 (97) | 0.94 (0.85, 1.00) | Excellent |
| C vs I | 62 (95) | 0.91 (0.80, 1.00) | Excellent |
| C vs J | 62 (95) | 0.91 (0.80, 1.00) | Excellent |
| D vs E | 63 (97) | 0.94 (0.85, 1.00) | Excellent |
| D vs F | 63 (97) | 0.94 (0.85, 1.00) | Excellent |
| D vs G | 63 (97) | 0.94 (0.85, 1.00) | Excellent |
| D vs H | 63 (97) | 0.94 (0.85, 1.00) | Excellent |
| D vs I | 62 (95) | 0.91 (0.80, 1.00) | Excellent |
| D vs J | 62 (95) | 0.91 (0.80, 1.00) | Excellent |
| E vs F | 63 (97) | 0.94 (0.85, 1.00) | Excellent |
| E vs G | 65 (100) | 1.00 (1.00, 1.00) | Excellent |
| E vs H | 63 (97) | 0.94 (0.85, 1.00) | Excellent |
| E vs I | 64 (99) | 0.97 (0.91, 1.00) | Excellent |
| E vs J | 64 (99) | 0.97 (0.91, 1.00) | Excellent |
| F vs G | 63 (97) | 0.94 (0.85, 1.00) | Excellent |
| F vs H | 63 (97) | 0.94 (0.85, 1.00) | Excellent |
| F vs I | 62 (95) | 0.91 (0.80, 1.00) | Excellent |
| F vs J | 62 (95) | 0.91 (0.80, 1.00) | Excellent |
| G vs H | 63 (97) | 0.94 (0.85, 1.00) | Excellent |
| G vs I | 64 (99) | 0.97 (0.91, 1.00) | Excellent |
| G vs J | 64 (99) | 0.97 (0.91, 1.00) | Excellent |
| H vs I | 64 (99) | 0.97 (0.91, 1.00) | Excellent |
| H vs J | 62 (95) | 0.91 (0.80, 1.00) | Excellent |
| I vs J | 63 (97) | 0.94 (0.85, 1.00) | Excellent |
| Across 5 Japanese readers | 61 (94) | 0.94 (0.86, 1.00) | Excellent |
| Across 5 non-Japanese readers | 62 (95) | 0.96 (0.89, 1.00) | Excellent |
| CI, confidence interval.  ^a^Readers A through E were non-Japanese and Readers F through J were Japanese.  ^b^The paired comparison was based on Cohen's Kappa statistics while the overall comparison was based on Fleiss' kappa statistic.  ^c^The kappa value is classified as follows: >70% to ≤80, good; >80% to ≤90, very good; > 90%, excellent. | | | |
